# Growing Up Under COVID-19: Young People’s Agency in Family Dynamics

**DOI:** 10.3389/fsoc.2021.722380

**Published:** 2021-10-05

**Authors:** Malika Shah, Sara Rizzo, Barry Percy-Smith, Leanne Monchuk, Enrica Lorusso, Chermaine Tay, Laurie Day

**Affiliations:** ^1^ Ecorys (United Kingdom), Birmingham, United Kingdom; ^2^ University of Huddersfield, Huddersfield, United Kingdom

**Keywords:** action research, civic participation, COVID–19, family dynamics, young people

## Abstract

The COVID-19 pandemic transformed the nature of family life in countries across the world. School, and workplace closures meant that families spent more time at home and had to confront new economic, social, and psychological challenges as a result of lockdowns and the greater proximity of family members. Policy, research and media coverage of the pandemic’s impact on family life has focused primarily on the economic costs borne by households. This article draws on the findings from an empirical research project funded by the UK Nuffield Foundation on “Politics, Participation and Pandemics: Growing up under COVID-19”, which worked with young people as co-researchers, to present an innovative perspective on the impact of lockdown on family relationships. The research team adopted a longitudinal ethnographic action research approach to document and make sense of the experiences of young people (aged 14–18) in four countries: Italy, Lebanon Singapore and the United Kingdom. The project used digital ethnography and participatory methods to track the responses of 70 young people to the challenges created by the pandemic. The study used the family as a prism for understanding how the lives of children and young people in different family circumstances and relationships were affected by the crisis. This article analyses, firstly, the complex shifting dynamics within households to identify the transformative effects of the pandemic on family life in various socio-cultural contexts. Secondly, it examines how young people’s agency shaped family dynamics. In conclusion, the authors recommend how the findings from the study can be used to inform government interventions designed to minimise the impacts of the pandemic on the social well-being and rights of children and young people, and to recognise them as active participants in family and civic life both during and after the pandemic

## Introduction

Government responses to the COVID-19 pandemic – school and office closures, new ways of working – transformed the nature of family life in countries across the world. Family members were forced to spend more time at home with close relations. Young people had to confront new economic, social, and psychological challenges associated with the pandemic and greater proximity to parents, carers and siblings.

A growing body of research based on survey data collected from adult participants has analysed the complex shifting family dynamics resulting from the pandemic in terms of the economic, social, and psychological challenges it created, as well as the individual and collective coping mechanisms adopted by families (Mariani et al., 2020; Prime et al., 2020; Salin et al., 2020). This article seeks to fill a gap in the literature by reporting on family dynamics from the perspective of young people using rich, longitudinal qualitative accounts of their experiences and behaviours.

The empirical research on which the article is based was funded by the Nuffield Foundation for the period April 2020 to September 2021, and carried out by Ecorys, an international research and consultancy company, and the University of Huddersfield, United Kingdom (Day et al., 2020). The project, entitled “Politics, Participation and Pandemics: Growing up under COVID-19”, was designed to provide in-depth qualitative insights into the impact of the COVID-19 pandemic on the lives of young people aged 14 to 18, and to make policy recommendations for promoting their well-being and rights during and after the pandemic. The project team were interested in how individual experiences of the pandemic were mediated through national, political, socio-economic, and cultural factors, intersecting with ethnicity, gender, sexuality, age, health conditions, and family/household living arrangements.

To explore these issues, the project team adopted a longitudinal ethnographic action research approach (Tacchislater and Hearn, 2003) to document, make sense of and communicate the changing experiences and behaviours of young people in Italy, Lebanon, Singapore, and the four nations in the United Kingdom. These countries were selected to reflect contextual differences in the political and cultural backdrop to the crisis, public health responses, and cultural orientations regarding child rights and political representation.

The rationale for the approach was to engage young people as co-researchers to learn from their lived experiences about diverse social realities in different national contexts according to their own terms of reference (Kirby, 2004). Involving young people as co-researchers in this project entailed young people being supported in actively undertaking their own research in line with their own priorities rather than the focus being prescribed by the adult researchers. This approach draws on principles of participatory action research (Percy-Smith et al., 2019b), specifically cooperative inquiry (Heron et al., 2006), in which research is approached as co-inquiry rooted in the everyday experiences of young people as participants (Abebe, 2009; Percy-Smith et al., 2019a). This article draws on qualitative data collected for the wider research project, using individual and group interviews with young people as co-researchers to communicate their critical reflections and sense-making from their individual and collective action research into the unfolding of the pandemic from their own perspectives.

In exploring the impact of COVID-19 on family life, the article considers family dynamics as a prism through which children and young people experienced the pandemic. It examines both the transformative effects of COVID-19 on family life across different national contexts, and the extent to which young people within their family contexts influenced the responses of family members to the pandemic. The authors conclude the analysis by drawing out the implications of their findings for development of both policy and theory.

## Review of the Literature

The article is framed by Bronfenbrenner’s (1979) socio-ecological systems model for understanding young people’s experiences in the context of family, community and state, as well as being mediated by the specificities and practices of family life (Edwards, 2020). The authors draw on family systems theory to understand the significance of interactions and relationships during the pandemic, as families continually shape, and are shaped by, their members and their interdependencies (Bowen, 1977). These concepts are not substantially developed in this article, which can primarily be viewed as providing empirical evidence in support of the model.

Research carried out in different countries by sociologists, psychiatrists, human geographers, social workers, economists, and epidemiologists has shown how family life was affected during the COVID-19 pandemic as a result of the loss of family members and anxieties associated with isolation, health concerns, increased unemployment, and financial vulnerability (Lebow, 2020). The increase in time spent at home dealing with these challenging circumstances led to family closeness and resilience as well as heightened family stress and conflict (Biroli et al., 2020, in Italy, the United Kingdom and the United States; Gadermann et al., 2021, in Canada; Lee and Ward, 2020, in the United States). Additional risks had to be faced by families with pre-existing vulnerabilities, including low socio-economic status, disability, underlying health conditions, family violence and conflict (Browne et al., 2015; Cluver et al., 2020, in South Africa). Increased difficulties were also reported due to disruptions to socio-cultural and religious traditions that are integral to family lives.

Research highlighting the centrality of the family in influencing the resilience of children and young people in the face of adversity suggests that children’s capacity to adjust to major shocks, such as a pandemic, is heavily contingent on family dynamics and relationships (Prime et al., 2020). Strong family relationships prior to the pandemic were found to act as a buffer against stress, facilitating sharing of responsibilities and providing the comfort of significant relationships. These ties offered much-needed social support at a time of loneliness (Mariani et al., 2020, in Italy) and provided opportunities for family time, especially leisure, and conversation (Salin et al., 2020, in Finland), to offset the negative effects of the pandemic.

Other authors have found that, even before the pandemic, young people were likely to be highly influenced by the emotions and outlook of family members. Those surrounded by family members with an optimistic outlook tend to display a “mirroring effect” (Gilbert, 2018, commenting on Bowen’s theory), whereby they experience similar positive feelings (Mariani et al., 2020). However, in the context of parental job and income losses, parents worried about the future and experienced depressive symptoms and negative interactions with their children (Jutengren, 2004, in Sweden; Kalil et al., 2020, in the United States), affecting their mental health (BBC Children in Need, 2020, in the United Kingdom).

Young people who reported the most positive experiences during the pandemic were those who were encouraged by their parents to adopt task-oriented coping strategies, which are said to be most appropriate in situations when the problem is “out of one’s control” (Mariani et al., 2020). These strategies included following a daily routine, refraining from constantly reading news and other updates about COVID-19, taking the opportunity to pursue hobbies, spending time outdoors, where possible, and using positive reappraisal/reframing of changing circumstances. Mutual reliance on family members during such challenging times has been found to strengthen family bonds, especially for low-income groups neglected by national welfare systems (Tang and Li, 2021, in China).

Earlier analysis of citizenship perspectives on family life highlighted the importance of “interdependence” rather than dependence of young people in relation to their parents, moulded by family solidarity and commitment manifest through young people’s “acts of citizenship” as participating subjects in society (Moosa-Mitha, 2005, in Canada). For example, other pre-pandemic literature documented the active contributions of children in the spheres of family, community, the economy, and inter-generational relationships (Abebe, 2019, in African countries; Esser et al., 2017, in Germany; Spittler and Bourdillon, 2012, in African countries). This work argued that young people are not passive victims of circumstance; instead, they possess “agency” in contributing to their own well-being and to that of their families, even in adverse or disadvantaged situations (Sorbring and Kuczynski, 2018). Whilst academic debates, rehearsed elsewhere, concerning the concept of young people’s agency (Esser et al., 2017; Spyrou, 2018; Abebe, 2019), seek to understand children’s agency in terms of context, structure, relationships and interdependence, in this paper the term is used to refer simply to young people’s own embodied sense of empowerment as social actors. In this respect, the article highlights and acknowledges the value of young people’s participation in its own right, rooted in their personal experiences.

Little attention has been paid in these many studies to young people’s agency and their contribution to family life during the COVID-19 pandemic, or to the implications of the pandemic on families as experienced from young people’s perspectives. The qualitative longitudinal ethnographic action research project, on which this study is based, was designed to engage participants as experts in their own lives and to provide rich insights into their everyday affective and social responses to the pandemic (Abebe, 2019; Percy-Smith et al., 2019b). This article seeks to show whether the earlier observations reported in the literature were confirmed by personal accounts of the experiences of the young people growing up under COVID-19 in different socio-cultural environments.

## Methodology

The empirical work described in this study was designed to analyse the intimate lives of young people from diverse backgrounds by engaging and supporting them as active participants in the research process. The approach adopted was qualitative, youth-centred and youth-led. The specific focus of the research was determined by the participants’ own circumstances and choices. The intention was not to extrapolate from the results to generate causal inferences at population level. Rather, elements of comparison and pooling were used to identify commonalities and differences within and across countries.

### Data Collection

The countries featured in the Nuffield project were selected to reflect different political, socio-economic and cultural conditions, and varying public health responses to the crisis. Italy was chosen as a country characterised by a liberal democracy, relatively high incomes, and a strong national culture of child rights, but limited youth representation in political decision-making. Italy was also chosen because it was the initial epicentre of the pandemic in Europe in March 2020. Although COVID-19 was concentrated in hotspots in the north of the country, central government quickly implemented a strict national lockdown followed by a three-tier system of restrictions.

The United Kingdom was selected for the study as a high-income country with a liberal democracy and youth representation in political decision-making, but a relatively high level of socio-economic inequality and a varying and inconsistent approach to child rights. To reflect differences across home nations, the research sample included young people from England, Northern Ireland, Scotland, and Wales. Compared to Italy, the United Kingdom was slower to be affected by, and to respond to, the pandemic. The government adopted a phased and then increasingly strict national lockdown, although the four nations subsequently varied in their implementation of the COVID-19-related restrictions.

As a small city state, Singapore is characterised by relatively low levels of inequality, and strong central government control. It was selected as an example of a Southeast Asian country with prior experience of managing epidemics with the SARS virus. The government succeeded in containing the pandemic in its early stages by imposing measures such as contact tracing using digital technology.

Lebanon represents a country in the Middle East and North Africa region with lower income status, high levels of income inequality and a large refugee population. The Lebanese political system is characterised by religious sectarianism, and the country has experienced decades of conflict resulting in a collapsing economy. With its relatively weak record on child rights, Lebanon has an ongoing history of youth protest and civil unrest. The Lebanese government’s response to the pandemic was based on containment. The government faced specific challenges due to the pre-existing economic crisis, poor internet connection, and the marginalisation of Syrian refugees, exacerbated by the Beirut port explosion in August 2020. The sectarian governance system further undermined an effective response to the pandemic (Di Peri, 2020).

All researchers and authors associated with the study acted in accordance with ethical and safeguarding standards observed by Ecorys and the University of Huddersfield. Ethical clearance was granted from respective national ethics committees prior to commencement of the work. Furthermore, the research was carried out in accordance with the legal and policy frameworks for child protection and safeguarding in each of the countries in the study. Just one safeguarding incident arose, which was resolved satisfactorily by the relevant child protection authorities within the country concerned.

An initial and ongoing assessment of the well-being of study participants was made, drawing on validated Child Outcomes Research Consortium (CORC) resources. This assessment involved conducting screener interviews with shortlisted young researchers, ensuring they had internet access and a private space where they could take part in calls. The screener interviews also assessed the young researchers’ health and well-being and whether their participation would be appropriate and would not cause any harm. Informed consent was sought and obtained from all research participants, and parental consent was obtained for young people under the age of 16. No issues were reported in obtaining parental consent, and parents were generally keen for their children to take part. All the young people participated voluntarily and were informed of their right to withdraw at any point in the study.

A second wave of semi-structured interviews was conducted by the adult researcher assigned to each of the panels. They sought to build on the trust and rapport that had been established in the early stages of the project, which provided an opportunity for the young people to reflect on and discuss their research findings in more depth, including observations and perspectives from family and friends, in addition to personal experiences in the first phase of the research. These interviews lasted between 70 and 90 minutes and included questions on young people’s views of the management of the pandemic as well as their own personal experiences. They were conducted in English, Italian, Arabic, and French by research panel leads who were native speakers, and the recordings were then transcribed and translated into English. The young people’s own research entailed associated ethical issues. As this article is primarily based on the young people’s interviews with the adult researchers, such ethical issues are not detailed here but can be found in the young researchers’ guide to action research (Ecorys and Huddersfield University, 2020).

### Sample and Participants’ Characteristics

Panels of ten young people aged 14 to 18 were recruited in each country, including the four constituent nations of the United Kingdom, through an application process: calls for participants were shared via social media, NGOs, public authorities, and organisations representing specific groups of young people, including Black Asian Minority Ethnic and Refugees (BAMER) and Lesbian, Gay, Bisexual, Transgender, Queer and others (LGBTQ+), as well as young carers. Applicants were invited to make a short written or audio/visual submission where they described their circumstances and experiences during the pandemic and their motivations for taking part in the study. A stratified purposive sampling framework was used, rather than random sampling, to shortlist applicants according to age, gender, ethnicity, specific health conditions, special educational needs or disabilities and different familial structures (Monchuk et al., 2020).

Whilst every attempt was made to achieve an equitable representation, the final decision about the selection of participants was determined by the need, given the aims of the study, to ensure young people with a variety of social, personal, and contextual characteristics were represented, irrespective of their nationality and gender. This decision was further justified by the need to ensure recruits had sufficient maturity and independence to engage actively, as co-researchers, with an online action research project lasting 18 months.

Young people were provided with ongoing training and mentoring throughout the research cycle. As incentives for participation, they were informed that they would receive a completion certificate at the end of the study, stating that they had been co-researchers on the project.

The project involved supporting 70 participants working in seven research panels to explore, reflect on and communicate the impact of the pandemic on themselves, their friends, family, and wider society through their own eyes. Eight of the original recruits opted out of the interview for a variety of personal reasons. The explosion in Beirut in August 2020, which occurred during the fieldwork period, accounted for four of the ten young people from Lebanon preferring not to take part in an interview. The article is based on the evidence from 62 semi-structured, online qualitative interviews carried out between July and September 2020.

As shown in Table 1, the sample of young people taking part in the study was skewed towards female participants, who constituted two-thirds of respondents (one young person identified as non-binary). The preponderance of female participants was explained by the fact that there was a greater volume of applications from girls and young women, meaning that they were correspondingly over-represented in the final sample. The decision was taken to recruit four panels within the United Kingdom, reflecting the status of the Nuffield Foundation as a UK funder and the priority to ensure a sufficient number and range of participants to explore UK policy responses to the pandemic with young people in requisite depth. The original intention was to secure equal representation across the home nations in the UK sample. In practice, the number of applicants from England was substantially higher, despite publicising the opportunity through networks in all four home nations.

**TABLE 1:** Participants’ characteristics.

	Italy	Lebanon	Singapore	United Kingdom	Total
Number of interviewees by country	11	6	11	34	62
Male	4	3	5	7	19
Female	8	3	6	26	43
Non-binary	0	0	0	1	1
Black and minority ethnic	3	5	3	7	18
LGBTQ+	1	0	0	4	5
Low income/socio-economic status	4	3	3	7	17
Children in state care	0	0	0	3	3
Specific health conditions	2	0	0	6	8
Special educational needs or disabilities	0	0	0	4	4

A limitation of the study was sample attrition, which is a common problem associated with longitudinal studies (Young et al., 2006). Attrition can be explained by the type of population targeted in the study (Boys et al., 2003) and the online nature of the research. Continuous interaction with young people over the 18-months duration of the project was compromised by poor internet connectivity, especially in Lebanon; “zoom fatigue” (Bailenson, 2021); and the need to rely on online platforms fully compliant with national General Data Protection Regulation (GDPR) legislation.

To maximise participant retention, researchers adopted tailored communication and engagement strategies to build up a strong rapport with the young people over time. These solutions included private messaging and one-to-one calls to check on participants and support them with the research, to enable ongoing discussion, and to allow for flexible engagement and the scheduling of calls around their other commitments.

### Data Analysis

In the first instance, in line with the action research approach adopted here, analysis was conducted by young people individually and collectively making sense of their own observations and those of their peers through diary entries, voice-notes, photography, art, and poetry, in collaboration with adult researchers. Adult researchers in turn undertook further analysis of findings according to the thematic research themes set out for the project, which included: family, friends and peer relationships, work and income, access to services, education, health and well-being, identity, and freedom of expression. The adult researchers adopted an inductive approach in analysing the data, guided by young people’s responses, and informed by ongoing dialogue with them. The data collected were imported into NVivo qualitative data analysis software and coded using a thematic approach (King et al., 2017).

This article draws on the semi-structured interviews and does not include analysis of the entirety of the data collected in the study. The interviews addressed a wide range of topics, not all of which were relevant to the central themes of this article. For example, participants’ views on actions taken by decision-makers and the public to respond to the pandemic, and recommendations about how to safeguard young people’s rights and well-being in the current and future pandemics were excluded. For the purposes of the article, the analysis was limited to data coded under the themes of Family, Impact of the Pandemic on Significant Others, Coping Mechanisms, and Identity and Beliefs.

To ensure that stakeholders were kept informed of the progress of the research and its policy relevance, the project team organised and attended online stakeholder events to support active engagement with the young people, to inform interventions and communicate recommendations for minimising the impacts of the pandemic on children’s well-being.

## Findings

While the research was not explicitly focused on family relationships, the study nonetheless revealed young people’s insights into the impact of the pandemic on family dynamics and their own roles in the family. In presenting the findings from the study about how young people’s family relationships were affected by the experience of growing up during the pandemic, the adult research team analysed the data for this article under three headings: the family as a prism mediating and colouring young people’s experiences of the pandemic; the transformative effects of the pandemic; and young people’s agency.

### The Family as a Prism

The findings from the study demonstrate that young people’s experiences of the pandemic were strongly influenced, in both positive and negative ways, by pre-existing family circumstances in terms of mental and physical health, education, socio-economic factors, and sexuality.

Across countries, it was evident that families with close and stable relationships found it easier to cohabit during the pandemic, and family dynamics were not significantly affected by the COVID-19 restrictions.

I’ve really enjoyed my family. We’ve always been really close anyway, so it’s just been lovely. So, to be fair it doesn’t really feel like it’s been that different from normal life, because we’ve always been near each other. (16, F, England)

This finding was especially true for young people where family income and parents’ work routines remained stable during the pandemic, and where parents could work from home, while still allowing them time to spend with the rest of the family at the end of the working day. Young people in these circumstances appreciated that their experiences of the pandemic and family dynamics were facilitated by socio-economic circumstances, and by living in spacious homes and with access to the physical and mental benefits of outdoor spaces.

Family dynamic is still really nice…we’re so lucky to live in the countryside where we can get out to the woods, or we have a decent sized garden, and just get outside, … Yes, that really helps. (18, F, England)

Across countries, young people who reported more positive experiences of the pandemic were those who could benefit from the optimistic attitudes of family members and collective coping mechanisms. Some parents were able to exert a positive emotional influence on young people by offering support during moments of anxiety or listening and talking to them when they needed a boost in morale or some advice on mental health.

My mum helped me a lot. She’s always optimistic. I think over those 3 months, she started to grow on me, like her way of thinking, like it’s okay, it’s going to be fine, it’s not the end of the world, and stuff like that. (16, F, England)

Many participants recounted how helpful they found their parents’ efforts to ensure they had a healthy routine and safe coping techniques. This support included meditation, self-care, time management and limited consumption of the news-cycle, which was even more important for young people with learning disabilities or those struggling with their mental health.

Her way of living and thinking really got to me. She introduced me to yoga, to meditation, because she’s a yoga teacher, but I’ve never had interest in doing yoga, but yes, she really helped me in that sort of sense. She helped to relax my mind a lot, and she also supported me a lot when I needed it, so I felt like because I was with my mum all the time, I was like, okay, it’s not going to get worse from here, I think it’s going to get better, so she was a big facilitating factor. (16, F, England)

Mum was very like, right, we start at nine o’clock, and then you have break then, and then … To keep it with school times, just because we’re both autistic, so it was easier to keep a routine going so it didn’t feel really weird, and then you got used to it after a while. They were just talking to me, trying to calm me down. (17, NB, England)

I think, if there’s one good thing that’s come out of COVID-19, it’s more of how not to distract myself, but to self-care. My mum and dad were really insistent on that. They were like: “You have to take better care of yourself.” (15, F, Singapore)

By contrast, young people who were surrounded by family members who were worried tended to worry more, which took a toll on their mental health. This concern was most visible in families from lower income backgrounds, especially in cases where parents were unable to work from home or had lost their jobs. Young people became increasingly afraid and worried when witnessing the difficulty of affording even basic needs such as rent. Despite measures like the Job Retention Scheme in the United Kingdom to protect employees from being made redundant, some young people were particularly concerned about the impact on their family’s main source of income or the gaps in government coverage, like sick pay, and the consequences this would have for the entire family. Within the sample, these effects were more visible among young people from Lebanon and those from low socio-economic backgrounds in the United Kingdom.

Our family’s economic situation changed. It is very bad. As a result of work stopping, it had been going down since the beginning of the quarantine and we don’t have any solution. We were all afraid of this instability. The economic situation was bad for my family and many other families since work stopped. I have been very sad since this started. (14, F, Lebanon)

A few young people were worried for their parents’ health because they were frontline workers, for example if social distancing or adequate Personal Protective Equipment (PPE) could not always be ensured, if they were in contact with COVID-19 patients, or if they had to work more than usual or for longer shifts.

I think it’s only 3 weeks ago he started slowly easing back into working, mostly because of the nature of his job. There isn’t really a way to socially distance inside a tiny car with people coming from Heathrow Airport from different countries and stuff like that. So, we were worried about his safety. (16, F, England)

Worrying about a family member’s health made young people worry more acutely about the pandemic. Some interviewees reported that seeing or knowing about a close relative catching COVID-19 represented “the lowest time” in the pandemic, either because they could directly witness the effects of the virus or because it functioned as a reality-check about the severity of the pandemic.

I found out that my aunt had coronavirus and I was just like, oh, this is kind of shocking … So that was the point where it hit me that this is not a joke anymore, this is for real. (16, F, England)

Many young people showed deep concern about the health of elderly family members, typically grandparents, and appreciated the additional difficulties that elderly people encountered during the pandemic. On the one hand, young people reiterated that the whole family was very conscious about restricting face-to-face contact with people from other households and maintaining social distancing as much as possible: most often only one family member would visit their grandparent(s) in their own homes. On the other hand, it was evident to young people how detrimental the lack of human contact and the fallout of a typical routine can be for older people:

I remember her telling me how she feels locked up. I remember that, because she’s not used to it, it’s sort of like a jail, you’re being jailed in your own house. (14, F, Lebanon)

They may want to go out and get groceries, as a small act of freedom or something. That was just really scary because we wanted them to stay as safe as possible. (15, F, Singapore)

Young people who did not have family members at risk reported being less worried. However, they noted their wider concern for the vulnerable in their communities and continued to follow all the necessary hygiene steps to prevent the spread.

Family life was most challenging for young people with caring responsibilities, especially in a few cases where a family member needed special care for mental health disorders. These young people faced heightened challenges as they had to adjust to sudden and unpredictable circumstances, particularly if a family member with special needs showed signs of distress. An Italian participant explained how an autistic family member had exhibited signs of stress and acted violently due to a lack of access to in-person therapy during the lockdown:

He couldn’t go to therapy, which he usually does two or three times a week, he couldn’t go to McDonalds. He actually became really violent, whether he was biting us or hitting us, because he was extremely stressed and unable to understand the circumstances and why he couldn’t go out. Luckily, he is only 8 years old. (18, M, Italy)

While none of the young people in the study reported violence or abuse, the experience of the pandemic and the lockdown proved particularly challenging for young people from the LGBTQ + community. Spending all the time at home was difficult for young people who felt their gender identity was not accepted. Family members could create a hostile environment for them, for example by calling a trans young person by their previous gender name. Other young people in this situation reported a significant loss of privacy and, in one case, a friend’s parent was said to be scrutinising their personal devices and discovered sensitive information about their child’s sexual identity and orientation. These young people found themselves trapped without the possibility of escaping to safer spaces like school or finding comfort in their friends’ company.

My friend is trans, his parents are not great with that. I saw him for the first time on Wednesday and he didn’t last 5 minutes in school. He started crying and had to leave. His parents are not okay with it. (15, F, Scotland)

Family life also significantly impacted young people’s experience of education during the pandemic. A few young people were deeply worried about their siblings’ academic performance, especially those attending university or practicing sports professionally. Nine young people across the United Kingdom, Italy and Singapore reported seeing their siblings’ or their own educational needs change due to the difficulty of sharing working spaces or devices with other family members.

We have to share a room, so we’d have to do it in the same room as each other. At one point I did just go out and sit on the trampoline, I’m like, sorry, I’ve had enough, I’m going outside and sitting on the trampoline and doing it. It was either that or the bathroom, or the kitchen floor. (17, NB, England)

These findings reveal the extent to which pre-existing family circumstances, family members’ outlook towards the pandemic and collective coping mechanisms mediated young people’s experiences.

### Transformative Effects of the Pandemic

For many young people, the COVID-19 pandemic changed and ultimately transformed family life. Whereas young people who enjoyed a stable family environment found that the pandemic reinforced this sense of closeness and unity, for others, family tensions and conflicts were exacerbated by living constantly in close proximity. Many young people in the study reported a deepening of family bonds and a renewal of family allegiances resulting from lockdown, school closures, and parents’ job losses. Examples were given of increased time spent with family members, including checking in more with each other, helping younger siblings with home-schooling and sharing communal activities and leisure time together, like watching television, playing boardgames, exercising, cooking, and eating together. In Singapore and the United Kingdom, for young people in higher income families whose lives were busy with extra-curricular activities before the pandemic, and whose parents usually worked long hours, COVID-19 created an opportunity to enjoy each other’s company more, particularly in cases where young people did not see much of their parents previously.

We could always be sitting outside together. We started watching box sets which we’d never done before, of different series on Netflix or something. That became, … it was just me and my parents, and that became our thing. We hadn’t had that before. (17, F, Northern Ireland)

My dad lost his job. I think it actually brought my family closer together because I think it was a blessing in disguise because usually, when my dad goes to work, he’s not home a lot and I don’t get to spend time with him. (14, F, Singapore)

In other cases, young people benefited indirectly from the switch to working from home, as they noticed their parents were less stressed thanks to the absence of commuting time.

I think she didn’t realise how much it stressed her out going to work and see, how stressful it is. I think then to see her not as stressed as usual, it was quite nice to see her more happy than she usually is. (14, F, Scotland)

Creating family routines was perceived by young people as helpful and functioned as a way to distract themselves from thoughts about what was going on outside the safety of the home, and to “make sure that there were positives to balance out the negatives” (15, F, Singapore). This led some young people to reassess their priorities and what is truly important to them, such as building a close relationship with younger siblings and enjoying a daily routine with their relatives. Having learnt to appreciate these moments, many young people expressed their willingness to preserve these even after the pandemic.

I saw my family members a lot more than I would see them prior to COVID-19. I would say that it’s a very gratifying experience, because I didn’t even realise how much time I was actually spending with my family in person … Now, I’m trying to rearrange all my activities such that I get to spend time with them. (17, M, Singapore)

Not all families saw enforced family togetherness as positive. Some experienced the opposite effect due to the increase in schoolwork at home, and the tendency for their parents’ work to spill over into home life.

Both my parents started to work from home. So, all three of us were stuck at home. I was in my own room and then my mum was in the living room and then my dad was in the study room. (16, F, Singapore)

“You can’t come in, I’m in a meeting.” I was like “Okay”. So now I wasn’t even allowed to go down to that bit of my own house, just kind of trapped in my room for the last 4 months. (17, F, Scotland)

Some young people reported their parents were so worried about the virus that they began practicing social distancing at home and stopped hugging, while grandparents renounced habitual walks with their grandchildren and other family rituals.

Inevitably, COVID-19 negatively impacted family traditions, particularly for migrant or religious families. Young people from migrant backgrounds who could not travel, for example to Greece, Malaysia, or Kurdistan, to see their relatives, resorted to Zoom to reduce the distance, or used their imaginations to relive memories from past travels. The most profound impacts were when they had to refrain from taking part in family traditions during religious festivals that used to make these moments special. This was particularly notable in Lebanon, as lockdown restrictions were imposed before Ramadan began and continued to Eid.

I know in Ramadan, especially, people have had to do a quarantine version of Ramadan. There were a lot of traditions, and every Islamic family has certain traditions they do on Ramadan, they have certain places they go to and everything. The coronavirus basically put a hold on that …. (14, F, Lebanon)

During the pandemic, for many young people, family life was characterised by increased tension and irritability. Young people and their parents widely reported feeling overwhelmed by daily COVID-19 news, bored and exhausted by the monotony of a restricted, sometimes stifling routine, and often intolerant of each other.

My mother and I kind of have conflicting personalities, so we had quite a few arguments, but my mum and my dad were both working. He kind of stayed out of my way a little bit, but tensions are high, everyone’s bored of lockdown, so you argue a lot and you can’t go anywhere. (15, F, Scotland)

At the same time, several young people recognised that such tension was understandable, and highlighted instead the strengthened family bonds that resulted from having gone through such a challenging experience together. This feeling was reported more often by respondents in Italy, probably due to the severity of Italy’s first wave and the fact that, at the time of the interviews, Italy had just exited a stringent lockdown.

We were never united as a group, whereas in the last few months we became very supportive of each other. Whenever one of us was feeling particularly down, someone else would step in and say: “Come on, we can make it!” (18, F, Italy)

The pandemic had a seismic impact on low-income families manifested in feelings of stress and, at times, hopelessness. Interviewees in the United Kingdom described their parents queuing at food banks and being unable to afford school costs, which left them feeling fearful and uncertain about their future. Some expressed concerns about their parents’ financial situation, heightened in single-income families with limited social benefits, where respondents were worried about the precarity of family finances if their working parent contracted COVID-19 and was unable to work. Others noted feeling increased pessimism and drew linkages between their parents’ reduced income and the longer-term impact of the pandemic on the global economy according to the family’s ability to respond.

I’m quite concerned about it because my dad, the pay from his new job isn’t really good, so I’m concerned about whether the economy will recover, and he will be able to find a better job. Also, for other people, I think our family is able to cope as we have savings, but what about those people who lost their jobs, and they have nothing left? (16, F, Singapore)

We really tried to cut our expenditure using these unnecessary ways, and only try to spend on what’s necessary for us, like food, water, cleaning supplies and everything, make sure that we can live properly, but live a basic life during the COVID period. (15, M, Singapore)

The transformative impact of COVID on family life, whilst being detrimental in many cases, simultaneously brought about a deeper sense of appreciation and resilience as families developed their own coping strategies. However, young people emphasised that coping mechanisms gradually lost efficacy over time, with tensions and irritability plaguing even the closest families as lockdown persisted. Tensions were heightened in lower income families, where family members were suffering from poor mental and physical health, and for LGBTQ + young people who felt alone and isolated.

### Young People’s Agency

A key finding cutting through these changing family dynamics was the importance of young people’s agency. In contrast to narratives of families in which parents are central figures who navigate hard times and provide for the family (Charles et al., 2008), the research reported in this article identified the key role young people played in influencing family responses to the pandemic. Young people faced new challenges due to lockdown restrictions and their parents’ shifting to working from home, as well as familial stress associated with caring responsibilities for younger siblings and older more vulnerable family members, and financial hardship. Such challenges compelled many respondents to assume greater responsibility for younger siblings at home and to play a more active role in family life. Their sense of responsibility was further heightened by reports that young people were much less likely to get seriously ill or die from COVID-19. Rather than being passive subjects within the family, participants in the study across all the countries presented themselves as custodians of their family’s health and well-being, as well as key actors in family decision-making. This response was particularly prevalent among the older participants in the sample.

In contrast to media presentations (Mercer, 2020; Rosney, 2020) of young people as reckless and cavalier in their interpretation of lockdown restrictions, the young people in the study expressed great concern and anxiety about safeguarding other family members and themselves from the virus. They were also worried about the increased stress and disruption generated by COVID-related restrictions.

I started to put on two masks and three masks and two gloves, just to take my own precautions and just please don’t talk to me or just keep social distancing. Especially, my dad, he has heart disease and respiratory problems, … so I don’t want him to get infected because I’m trying to do something for my community. (16, F, Lebanon)

Spending more time at home resulted in young people becoming more aware of the extent of their parents’ responsibilities. They were able to witness close-up how much their parents did for the family unit on a daily basis. This newfound appreciation fed into a heightened sense of understanding, closeness, and solidarity with their family and of their own agency as a family member.

All these things make you much more appreciative of your parents. When I look after my little siblings or when I do something, I just feel much more appreciative of what they do in the day. That has led me to become much closer to them. (17, F, England)

I will help them with some stuff, and they would help me with some stuff, so it made us more like interconnected and interdependent in a sense. (16, F, Singapore)

Some young people became more aware of what they could do to reduce the pressure on their parents and offset the adverse consequences of the pandemic. One young woman reported taking on a more involved caring role for her mother, who had a health condition. During lockdown, she became much more aware of the difficulties her mother faced and tried to support her as much as possible. Other young people in Italy and the UK reported taking on increased responsibilities, such as home-schooling and caring for their younger siblings.

I have my little brother, who was getting work, but it was just papers sent home with no guidance or help from teachers. So, I had to teach my little brother because my parents aren’t really good with English, so they can’t really translate what they know in Kurdish into English, so I have to do all the teaching for him. (16, F, England)

Another young person described making decisions with her parents and taking collective action to support the youngest child in the family who is neurodivergent. The young woman was not aware of how emotionally draining it was for her parents to take care of her sister until the pandemic.

I think as a family we had to make decisions together − of course we were following the rules of the government − but make decisions as to what was best for us as a family. So, my sister, who is autistic, to be honest, before the pandemic she didn’t go out the house very much either. She didn’t go to school at all because she was unable to go into mainstream school. (14, F, England)

Interviewees also recognised their limitations in helping younger siblings with their feelings of loneliness, mental health issues and increased school pressure during the pandemic.

I live with him and I see how much COVID has impacted him [younger brother], and it’s really heart breaking when you can’t really do anything about it, because I’m not a trained professional…We try our best to help him, but there are things that we can’t help him with. (17, F, Northern Ireland)

In reconfiguring family relationships through their interaction with family members in the home, young people became more aware of their own and others’ views and perspectives:

I got to know my dad better, especially in terms of his political views, which turned out to be different than what I thought. I heard him comment on the news sometimes, and I would stare at him thinking “What are you saying?” and this would trigger a discussion. (17, F, Italy)

This sense of growing confidence in challenging their parents’ political views was echoed by a young woman of the same age in Scotland. She found that the pandemic gave her the opportunity to engage with and challenge her father’s views, which were very different from her own:

I spent some time with my dad, he has a lot of very far-right beliefs, and I do not share any of those, so I think I’ve become more opinionated and vocal on those, and standing up to him, and challenging his far-right beliefs. (17, F, Scotland)

These comments show how COVID-19 offered young people an opportunity to get to know their family members better, in terms of their attitudes, “likes and dislikes” as well as political views. For a few participants, the pandemic heightened their awareness of contrasting, and sometimes surprising, political opinions held by their parents, since they had never previously had the opportunity to discuss politics with them.

## Discussion and Conclusions

The findings from the study reported in this article suggest that, despite the lack of comparable evidence from all four countries, young people reacted to, and were affected by, the COVID-19 pandemic in similar ways. Everywhere, participants recounted “stories of heroic family closeness and resilience” as well as “unmitigated family stress and conflict” (Lebow, 2020, p. 310). Through the voice of young people, the findings support the evidence outlined in the literature review that young people’s resilience to adversity is largely determined by their family dynamics and relationships.

Socio-economic status, disability and health conditions, pre-existing ties as well as parents’ attitudes were found to be crucial in mediating young people’s experiences of the pandemic. Collective and task-oriented coping strategies adopted by families accounted for the most positive experiences of the pandemic among young people. Other variables considered in the wider study, such as political and cultural contexts, proved less critical in helping young people to cope. The findings suggest that young people cannot be considered in isolation to their families. Rather, they are embedded within complex family structures, which shaped their reactions and responses to the pandemic.

The active participation of young people as co-researchers – engaged not just as interviewees but also as close observers and commentators − was an original feature of the study. It allowed the research team to confirm the importance of the role played by young people’s agency within the family, in influencing family responses to the pandemic, and broadening perspectives on young people’s participation in everyday contexts (Esser et al., 2017; Spyrou, 2018; Abebe, 2019; Percy-Smith et al., 2019a). Their agency was illustrated by their decisions to take on a more active caring role and by challenging their own beliefs and sense of identity in relation to those of other family members. These changing relationships between young people and family members emphasise the significance found in earlier work of family as a site for civic and citizenship engagement and the realisation of the importance of interdependence (Cockburn, 1998), rather than relationships of dependence within families (Wyn et al., 2011).

Important aims of the project were to create qualitative evidence of how children and young people were experiencing and making sense of COVID-19 that could be used to inform policy. The findings reported in this article suggest that policy responses designed to promote the well-being and development of young people should consider wider familial contexts and prioritise targeted support for families with specific needs, whilst recognising young people as autonomous citizens who are active participants in both family and civic life. In this respect, we can understand the significance of socio-ecological contexts of young people’s lives as a fluid two-way process of dynamic interaction rather than linear contextual influences. In so doing, our empirical research contributes to family studies theories by reinforcing the significance of Bowen’s (1977) theory of family as a complex system of social interactions, but which play out in different ways according to different cultural contexts.

## Data Availability

In accordance with the project’s privacy policy and ethical consent requirements provided by participants, the data presented in this article are not readily available. Requests to access the datasets should be directed to sararizzo@ecorys.com.
